# Enhanced Photoelectrochemical Property of TiO_2_ Nanotube Array Photoanode Deposited with Al,Cr-Codoped SrTiO_3_ Nanocubes

**DOI:** 10.1021/acsomega.3c08014

**Published:** 2024-01-03

**Authors:** Shiho Hamazaki, Kazuki Inoue, Atsunori Matsuda, Go Kawamura

**Affiliations:** Department of Electric and Electronic Information Engineering, Toyohashi University of Technology, Toyohashi, Aichi 441-8580, Japan

## Abstract

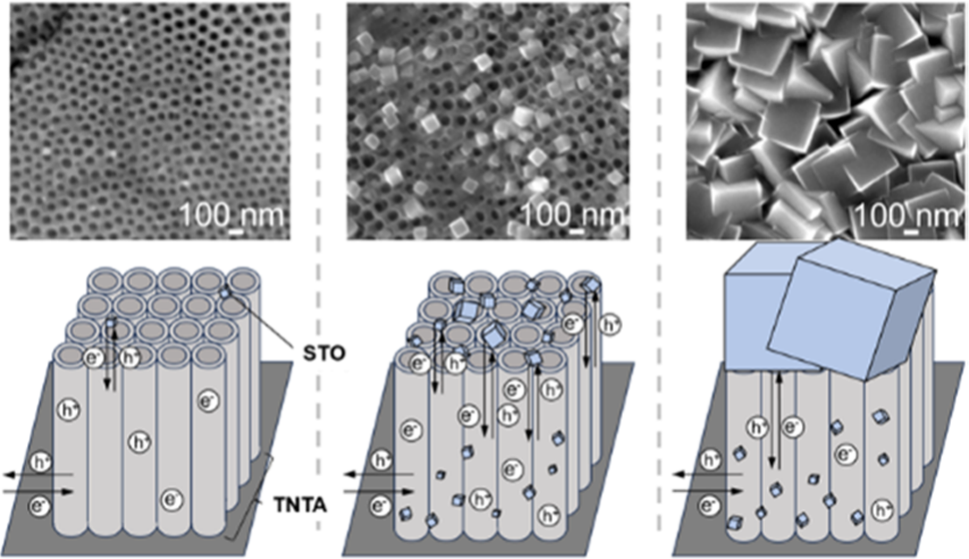

There is a demand for the effective utilization of solar energy with highly functional photoelectrodes for photoelectrochemical (PEC) applications, such as water splitting and CO_2_ reduction. TiO_2_ nanotube arrays (TNTA) with a large surface area have been studied as potential photoelectrodes mainly due to their strong oxidation potential. However, it has disadvantages of fast charge recombination and little responsivity to visible light. In this study, we prepared TNTA by anodizing a Ti plate and decorated the TNTA with Al,Cr-codoped SrTiO_3_ (STO) nanocubes through a hydrothermal treatment to enhance the PEC properties. We also prepared pristine and undoped STO-decorated TNTA for comparison. The hydrothermal treatment duration was optimized for the TNTA-STO:Al,Cr sample to achieve the best PEC performance. Finally, the possible PEC reaction mechanism was proposed based on the obtained experimental results.

## 1 Introduction

Energy and environmental issues have gained importance in recent years. Global primary energy consumption (FY2021)^1^ shows that fossil fuels account for 82% of the total consumption. Fossil fuels emit greenhouse gases and are the major cause of global warming. One countermeasure is the use of renewable energy. Renewable energies are clean energy sources such as solar and wind power that do not deplete and do not emit greenhouse gases. H_2_ is attracting attention as a next-generation energy source to replace fossil fuels. Unlike fossil fuels, H_2_ does not emit greenhouse gases when burned. There are several types of H_2_. Green H_2_ is produced by using renewable energy sources, such as solar power, in a production process that emits no CO_2_. Gray H_2_ and blue H_2_ are obtained by transforming fossil fuels to produce gas and then extracting H_2_ from the gas. H_2_ is generally produced by steam reforming,^2^ which reforms methane and other substances to produce gray H_2_, and is already widely used in industry, but the problem is that the production process emits CO_2_.^3^

In order to avoid CO_2_ emissions, green H_2_ must be generated from renewable energy sources such as solar and wind power.^3^ Electrolysis, photocatalytic (PC) water splitting, and photoelectrochemical (PEC) water splitting, which use redox reactions, do not emit CO_2_. Electrolysis is performed by applying an external bias to electrodes in an electrolyte solution and requires a large amount of electricity, and the equipment in practical use itself is expensive. PC water splitting is a low-cost method in which fine semiconductor powder is dispersed in water or placed on a panel and irradiated with light, but it is driven without an external bias and has a low efficiency. PEC water splitting has the advantage of high conversion efficiency from light energy to chemical energy, which can partially compensate for the large power required for electrolysis. Therefore, PEC water splitting, which can produce H_2_ directly from water using a redox reaction with sunlight, a renewable energy source, is attracting attention. Upon light irradiation, a semiconductor photoelectrode absorbs photons with energy above its band gap energy, causing electrons to be excited to a higher energy state (conduction band) and leaving holes where the electrons left off (valence band).^4^ By applying an external bias, the excited electrons generated at the anode (semiconductor photoelectrode) move to the cathode side through an external circuit and reduce the water/proton to produce H_2_. The remaining holes oxidize water to produce O_2_ at the anode side. When photoelectrodes are used for PEC water splitting, they should have an optical function for maximum absorption of solar energy and a low overpotential/catalytic function for water splitting.^3^

TiO_2_ has been used for PEC water splitting research^5^ due to its advantages such as high chemical stability, strong oxidation potential,^6^ and nontoxicity. In water splitting, the oxidation of water requires the potential of the valence band to be more positive than the redox potential of O_2_/H_2_O, 1.23 V vs NHE (pH = 0), and the reduction of water needs the potential of the conduction band to be more negative than the redox potential of H^+^/H_2_, 0 V vs NHE,^4^ and TiO_2_ satisfies these condition. In order to increase the surface area for enhanced PEC performance, TiO_2_ nanotube arrays (TNTA) are often fabricated by the anodization of a Ti foil as a photoelectrode. The morphology (tube length, pore size, wall thickness, etc.) is easy to get controlled by changing the anodization conditions such as voltage, temperature, time, electrolyte composition, pH, electrode type, etc.^7−9^ The surface of TNTA can be decorated with perovskite-type MTiO_3_ (M = Pb, Ba, Sr, Zn, Co, Ni, Ca) nanocrystals by hydrothermal treatment of TNTA in appropriate cation-containing aqueous solutions in order to decrease charge recombination rate/overpotential and increase visible light response.^10−13^ Among the perovskites for decoration, SrTiO_3_ (STO) is one of the most studied materials since the photogenerated charges in the system are effectively transferred among STO, TiO_2_, and Ti foil, resulting in high PEC performance under light irradiation.^14−18^ STO has a more negative lower end of the conduction band than TiO_2_ and can produce O_2_ and H_2_ by water splitting under UV irradiation, similar to TiO_2_. STO itself recently showed nearly 100% photocatalytic solar-to-H_2_ conversion efficiency when it was doped with Al and decorated with appropriate cocatalysts, which were Rh/Cr_2_O_3_ and CoOOH.^19^ This further suggests that STO should be a high-potential material for the water splitting application. When STO and TiO_2_ are combined, electron transfer from the conduction band of STO to the conduction band of TiO_2_ and hole transfer from the valence band of TiO_2_ to the valence band of STO occur under UV irradiation, and the separation of photogenerated electrons and holes is promoted, thereby improving photocatalytic performance.^20^ The main drawback of the STO and TiO_2_ system used under solar irradiation is their large band gap energy (∼3.2 eV), which is unable to absorb visible light. Since 3.2 eV corresponds to a wavelength of about 400 nm, it can only absorb UV with wavelengths below 400 nm and is unlikely to react with visible light, which is a major component of sunlight. Therefore, it is necessary to extend the absorption wavelength to the visible light region to improve the PEC performance under sunlight. Cr, F, or La doping has been carried out by adding these ions in the aqueous solution for hydrothermal treatment for controlling the electronic structure of STO to add visible light response.^21−23^ These doped STO-modified TNTA have shown high PEC performance and would have the capability for further improvement.

In this work, TNTA decorated with Al,Cr-codoped STO nanocubes (TNTA-STO:Al,Cr) were prepared by an anodization process and successive hydrothermal treatment, and their PEC properties were evaluated. As stated above, Cr doping would introduce visible light response to STO. The Al doping, on the other hand, would reduce the charge recombination in STO.^26^ Furthermore, codoping of Al and Cr to STO has never been reported so far. The hydrothermal treatment duration was altered to find an appropriate deposition amount and size of STO. Finally, the PEC reaction mechanism was discussed based on the doping effects and the conditions of deposited STO by PEC measurement and structural investigation results.

## 2 Experimental Methods

### 2.1 Sample Preparation

Disordered TNTA were formed by performing the first anodization using a Ti foil as the anode and a Pt rod as the cathode. A DC voltage of 60 V was applied for 2 h in an electrolyte at 20 °C. After preparing the disordered TNTA, they were washed with ion-exchanged water (IEW), and ultrasonic treatment was performed for 1 h in IEW to remove the formed disordered TNTA and obtain periodic nanovoids on the Ti foil. Next, a 12 mm diameter circle mask was placed on the Ti substrate with nanovoids, which will be used as the anode for the second anodization. A pristine Ti foil was employed as the cathode. A 30 V DC voltage was applied for 30 min in an electrolyte at 53 °C for the second anodization. After the second anodization, the Ti foil was left soaked in the electrolyte for 8 min with agitation at 270 rpm at 53 °C to remove C-doped TiO_*x*_ layer from the TNTA surface.^25^ Different electrolytes were used for the first and second anodization. For the first anodization, an electrolyte containing IEW (1.24 M) and NH_4_F (0.09 M) in ethylene glycol (EG) was used, and for the second anodization, a mixture of EG and dimethyl sulfoxide (DMSO) prepared at a volume ratio of 1:1 (vol %) with IEW (0.1 M) and NH_4_F (1.5 M) was employed. The prepared TNTA was heat-treated by placing it in an electric furnace at 500 °C (ramp rate: 3 °C/min) for 1 h.

The obtained TNTA was then placed inside an autoclave reactor filled with an aqueous mixture of Sr(OH)_2_ (5.1 mM), Al(NO_3_)_3_ (0.21 mM), CrO_3_ (0.32 mM), and NaOH (3.1 M) with the TNTA surface facing downward. The downward setting was implemented to protect TNTA from precipitation during hydrothermal treatment. The autoclave containing the mixed aqueous solution and TNTA was placed in an electric furnace at 150 °C and hydrothermally treated for predetermined times. The autoclave reactor was then cooled under running water for approximately 5 min before the sample was removed and washed with IEW. Finally, TNTA-STO:Al,Cr were obtained by drying at 60 °C for 1 h in a drying oven. For comparison, TNTA-STO without doping was also prepared by simply excluding the raw materials of Al and Cr elements from the aqueous solution for hydrothermal treatment.

### 2.2 Structural Characterization

The morphology of the prepared samples was observed by using a scanning electron microscope (SEM, S-4800, Hitachi High-Tech, Japan). The crystal structure was analyzed using an X-ray diffractometer (XRD, Smart Lab, Rigaku, Japan) with a Cu Kα irradiation line. The elemental composition and chemical state were analyzed by using an X-ray photoelectron spectroscope (XPS, PHI Quantera SXM-CI, ULVAC Phi, Japan).

### 2.3 PEC Characterization

Electrochemical measurements were performed by using the fabricated sample as the anode, a Pt coil as the cathode, and a saturated calomel electrode (SCE) as the reference electrode in a three-electrode system. A 0.2 M Na_2_SO_4_ + methanol (10 vol %) solution was used as the electrolyte, and simulated sunlight and visible light were used as light sources. The irradiation intensity for each light source was 1 sun (100 mW/cm^2^) for simulated sunlight (AM1.5G filter built-in Xe lamp) and 1 sun for visible light (a short wavelength cut filter was used: 422 nm). The applied voltage range for the photocurrent measurement was −0.7 to +0.7 V vs SCE (this equals 0–1.4 V vs reversible hydrogen electrode (RHE) when pH is 7). The frequency range for the PEC impedance spectroscopy (PEIS) measurement was 10 mHz to 1 MHz. The light source for the PEIS measurement was also simulated sunlight with 1 sun intensity.

## 3 Experimental Results

### 3.1 Structural Characterization

**Figure 1:**
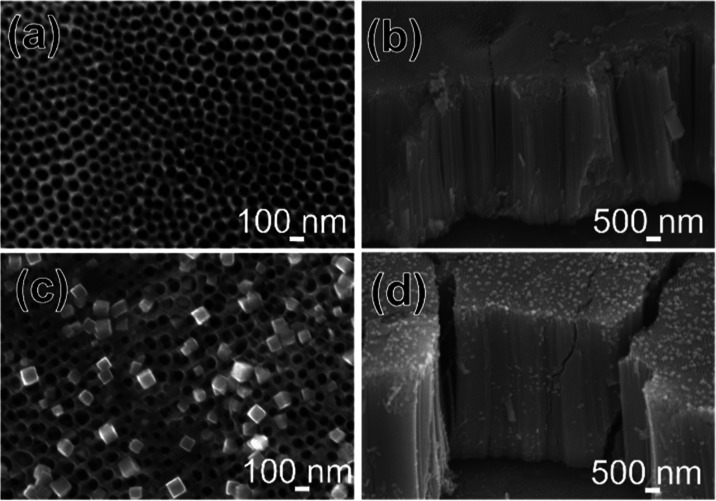
SEM images of TNTA (a: top; b: cross section) and TNTA-STO:Al,Cr_1 h (c: top; d: cross section).

Figure 1 shows the SEM images of the TNTA (a and b) and TNTA-STO:Al,Cr prepared by the hydrothermal treatment for 1 h (TNTA-STO:Al,Cr_1 h) (c and d). The pore size and tube length of TNTA were approximately 50–100 nm and 7.5 μm, respectively, which were unchanged by the hydrothermal treatment. On the other hand, STO nanocubes with sizes of 50–125 nm were deposited on the TNTA surface after hydrothermal treatment. The morphologies of the samples with and without Al and Cr doping were the same.

**Figure 2:**
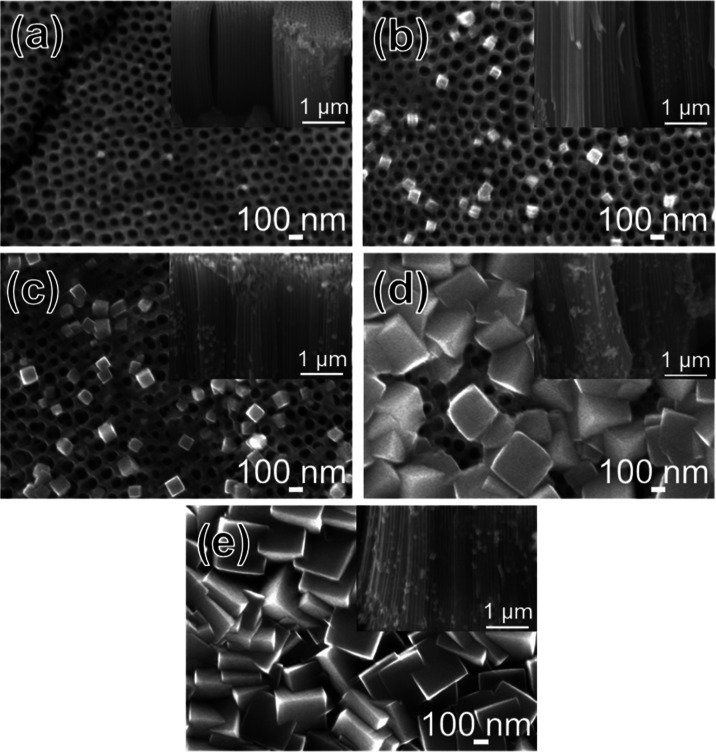
SEM images of TNTA-STO:Al,Cr (top and cross section) prepared by varying the duration of hydrothermal treatment: (a) 0.5 h, (b) 0.75 h, (c) 1 h, (d) 1.25 h, and (e) 1.5 h.

Figure 2 displays the SEM images of TNTA-STO:Al,Cr prepared by hydrothermal treatment for different durations ranging from 0.5 to 1.5 h. The amount of STO nanocubes on the surface of TNTA increased with the duration of the hydrothermal treatment. The STO size also increased up to 380 nm, which is observed on the top of TNTA-STO:Al,Cr_1.5 h. STO nanocubes are also seen in the inserted cross-sectional images, and the amount of STO on the cross section increased with the duration of hydrothermal treatment. The size of the STO on the cross section increased up to 130 nm, which is observed in TNTA-STO:Al,Cr_1.5 h. The amount and size of STO did not increase when the hydrothermal treatment duration was elongated to 2 h. This explained that the formation and growth of STO stopped at around 1.5 h; i.e., with the shorter hydrothermal duration, the STO nanocubes were still in the process of growing, which is why the size of STO was smaller than the 1.5 h sample.

**Figure 3:**
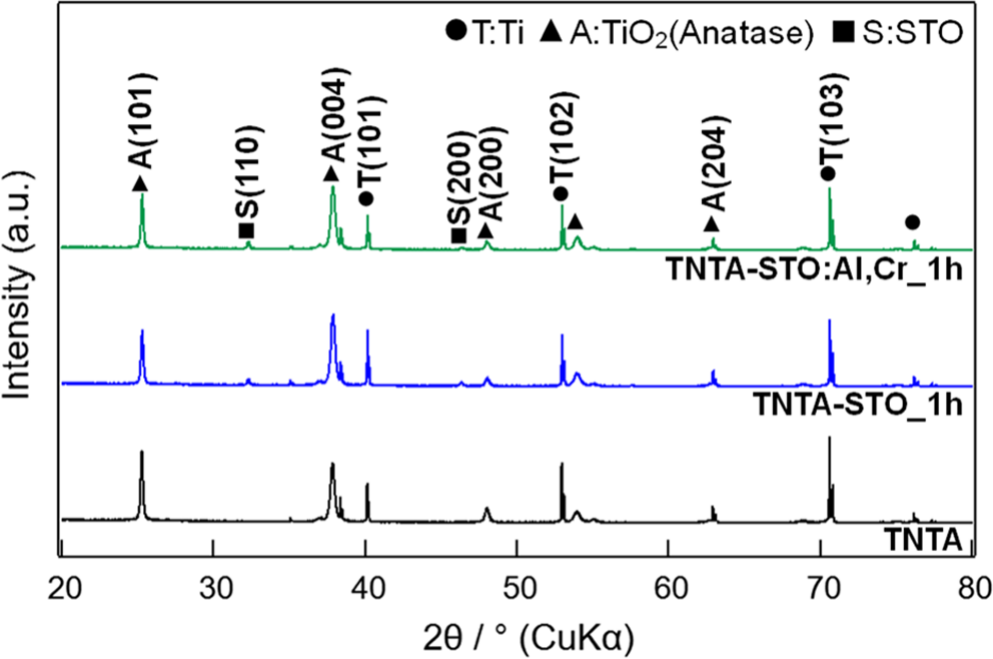
XRD patterns of TNTA, TNTA-STO_1 h, and TNTA-STO:Al,Cr_1 h.

Figure 3 presents the XRD patterns of TNTA, TNTA-STO_1 h, and TNTA-STO:Al,Cr_1 h. In all samples, diffraction peaks characteristic of metallic Ti (JCPDS: 44-1294) and anatase TiO_2_ (JCPDS: 73-1764) were observed. Besides, a few peaks corresponding to perovskite STO (JCPDS: 35-0734) were also found in TNTA-STO_1 h and TNTA-STO:Al,Cr_1 h samples. These XRD and SEM results indicate the successful fabrication of uniform TNTA with and without STO nanocubes on them. However, as the XRD peaks of STO were quite small, peak shifts confirming the Al and Cr doping could not be evaluated using the XRD data. The Raman spectra were also measured; however, only anatase peaks appeared because of the low quantity of STO (Figure S1).

**Figure 4:**
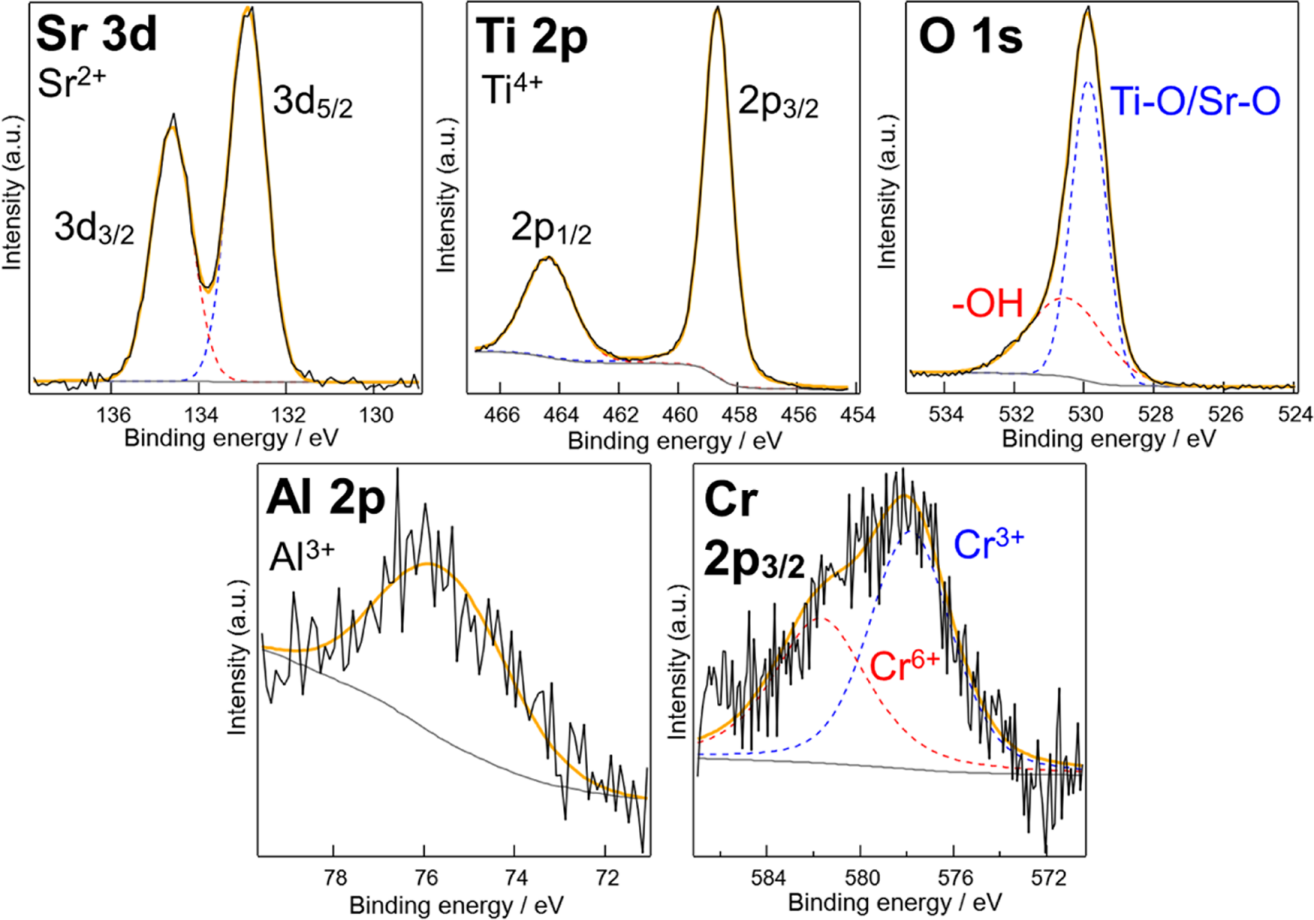
XPS analysis of TNTA-STO:Al,Cr_1 h.

Figure 4 illustrates the XPS analysis of TNTA-STO:Al,Cr_1 h, and Table 1 presents the corresponding quantitative results expressed in atomic percentage (atom %). The elemental analysis was also attempted by energy-dispersive X-ray spectroscopy, but a strong signal from the substrate made the analysis difficult (Figure S2). The Ti 2p and Sr 3d peaks in Figure 4 indicate the predominant presence of Ti^4+^ and Sr^2+^,^26^ which are the main components of TiO_2_ and STO. In general, Ti^3+^ is often observed in STO owing to its n-type nature, while there was little Ti^3+^ in our sample. This indicates the successful doping of Al^3+^, which reduces Ti^3+^ and oxygen vacancy in STO.^24^ Two distinct peaks are observed in the O 1s region, where a minor peak at near 532 eV corresponds to hydroxyl groups on the surface, and a main peak at near 530 eV corresponds to O atoms bonded to Ti and Sr.^27^ The Al 2p peak appeared at around 76 eV,^28^ which aligns with Al^3+^ derived from the oxide.^29^ A smaller Cr peak observed near 582 eV corresponds to Cr^6+^ and a larger peak near 578 eV corresponds to Cr^3+^.^30−32^ This is reasonable because doping STO with Cr basically results in the replacement of Ti^4+^ by Cr^3+^, leading to the formation of Cr^6+^ and oxygen defects as minor components.^33^ These XPS analyses provide conclusive evidence of Al and Cr doping into STO. The reason why the atom % of Ti was relatively larger than Sr is explained by the fact that TNTA surface was not fully covered by STO, which was proved, for example, by Figure 2c. The successful Cr doping was also confirmed by the UV–visible spectra of the samples where TNTA-STO:Al,Cr_1 h showed increased absorbance in the visible region compared to TNTA-STO_1 h (Figure S3).

**Table 1:** Quantitative Elemental Analysis Results (atom %) of TNTA-STO:Al,Cr_1 h

sample	Ti	O	Sr	Al	Cr
TNTA-STO:Al,Cr_1 h	21.4	69.9	5.8	1.1	1.8

### 3.2 Photoelectrochemical Characterization

**Figure 5:**
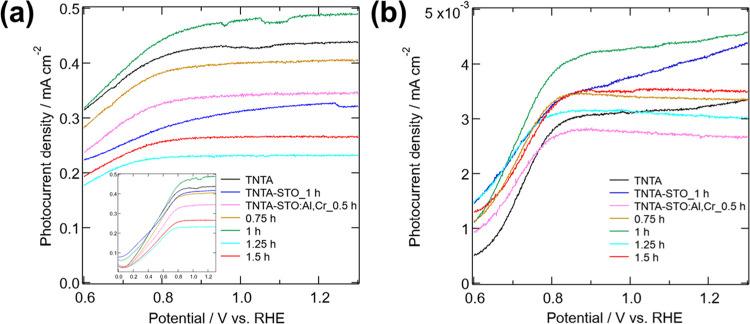
LSV of prepared samples under (a) simulated sunlight and (b) visible light.

Figure 5 shows the linear sweep voltammogram (LSV) of TNTA, TNTA-STO_1 h, and TNTA-STO:Al,Cr with various hydrothermal treatment durations ranging from 0.5 to 1.5 h. Figure 5a,b shows LSV measured under the illumination of simulated sunlight and visible light, respectively. The dark current measured before the photocurrent measurements is also shown in Figure S4. Notably, TNTA-STO:Al,Cr_1 h exhibited the highest photocurrent density in both figures. However, the decoration of TNTA with doped and undoped STO resulted in a decrease of the photocurrent value in some cases. This is totally opposite from the initial idea that the decoration reduces charge recombination and increases visible light absorption, leading to enhanced PEC performance, as described in detail in the Section 1. This performance deterioration is presumably due to the following reasons. In the shorter hydrothermal duration cases, the degree of STO crystallinity is too low; thus, STO would contain many defects that work as charge recombination centers. On the other hand, longer hydrothermal treatment resulted in the formation of large STO cubes covering the pores of TNTA, which reduces the surface area of the photoanode, leading to the lowered PEC performance. It is worth mentioning here that longer hydrothermal treatment would have some merit as well, for example, higher crystallinity, better electronic contact at interfaces, etc. Thus, the final PEC performance is determined by the balance of several factors. LSV measured without methanol in the electrolyte was also recorded (Figure S5). The results showed the same tendency as the case using methanol; that is, TNTA-STO:Al,Cr_1 h showed improved photocurrent whereas too short or too long hydrothermal duration resulted in low photocurrent. This proves that both electrolytes can be used to investigate the property of photoanodes without critical difference, while the addition of methanol leads to larger and stable photocurrent, which makes the discussion more reliable. In order to further investigate the PEC properties of the samples, the PEIS was measured.

**Figure 6:**
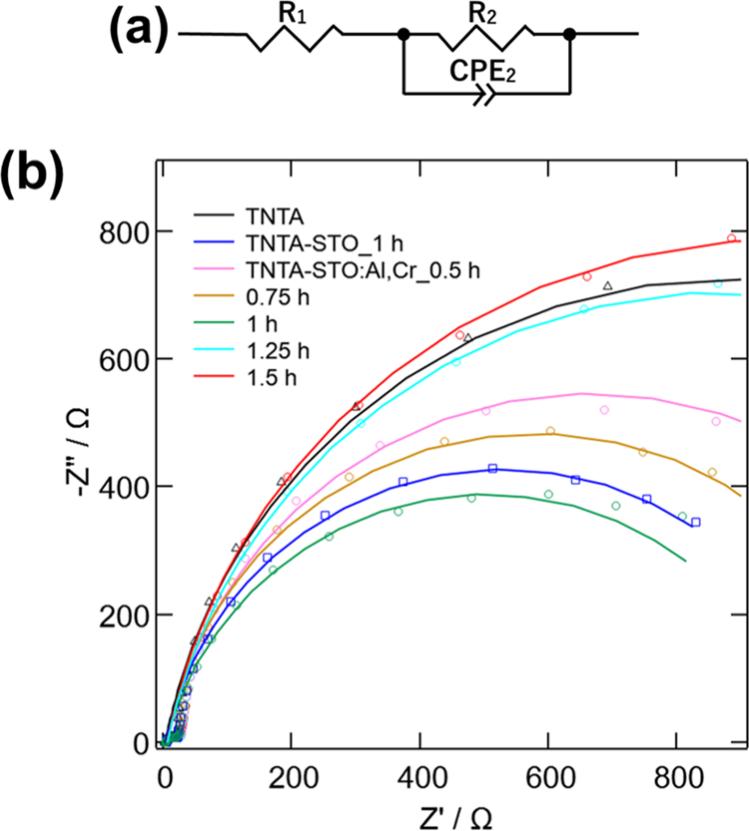
(a) Equivalent circuit and (b) Nyquist plots of TNTA, TNTA-STO, and TNTA-STO:Al,Cr.

Figure 6a shows the equivalent circuit employed for fitting the PEIS data. Here, *R*_1_ represents the internal resistance and *R*_2_ corresponds to the photoanode/electrolyte charge transfer resistance.^34^ Constant phase element (CPE) is a circuit element used to represent the capacitive semicircular strain. Replacing the double-layer capacitance with a CPE reflects the inhomogeneity of the surface layer and improves the adequacy of the fitting.^35^ Therefore, CPE_2_-T and CPE_2_-P correspond to the double-layer capacitance between photoanode/electrolyte. Figure 6b shows the Nyquist plots of the samples with fitting curves, and Table 2 shows the calculated parameters from the PEIS analysis. Compared to *R*_2_ values, *R*_1_ values are negligibly small, ranging in the order of a few ohms. Therefore, it is obvious that *R*_2_ is the main factor limiting the PEC performance of the samples. Among the obtained *R*_2_ values, TNTA-STO:Al,Cr_1 h possesses the smallest resistance of 1000 Ω, indicating a reduced charge recombination rate. This is presumably due to the moderate amount and size of deposited STO in the sample; that is, STO deposition results in the ideal formation of type-II heterojunction with TiO_2_ and increases surface area as mentioned above. Based on the results obtained from the LSV and PEIS analyses, it can be concluded that TNTA-STO:Al,Cr prepared by 1 h hydrothermal treatment exhibits the best PEC performance which is due to a lower charge recombination rate and higher active surface area.

**Table 2:** PEIS Parameters Calculated from the Nyquist Plots

sample	*R*_1_, Ω	*R*_2_, Ω	CPE_2_-T	CPE_2_-P
TNTA	3.0	1750	0.032	0.88
TNTA-STO_1 h	3.9	1050	0.036	0.87
TNTA-STO:Al,Cr_0.5 h	6.0	1320	0.030	0.88
0.75 h	4.5	1150	0.033	0.89
1 h	3.6	1000	0.028	0.84
1.25 h	6.1	1730	0.028	0.87
1.5 h	5.8	1900	0.027	0.88

### 3.3 Mechanism Discussion

**Figure 7:**
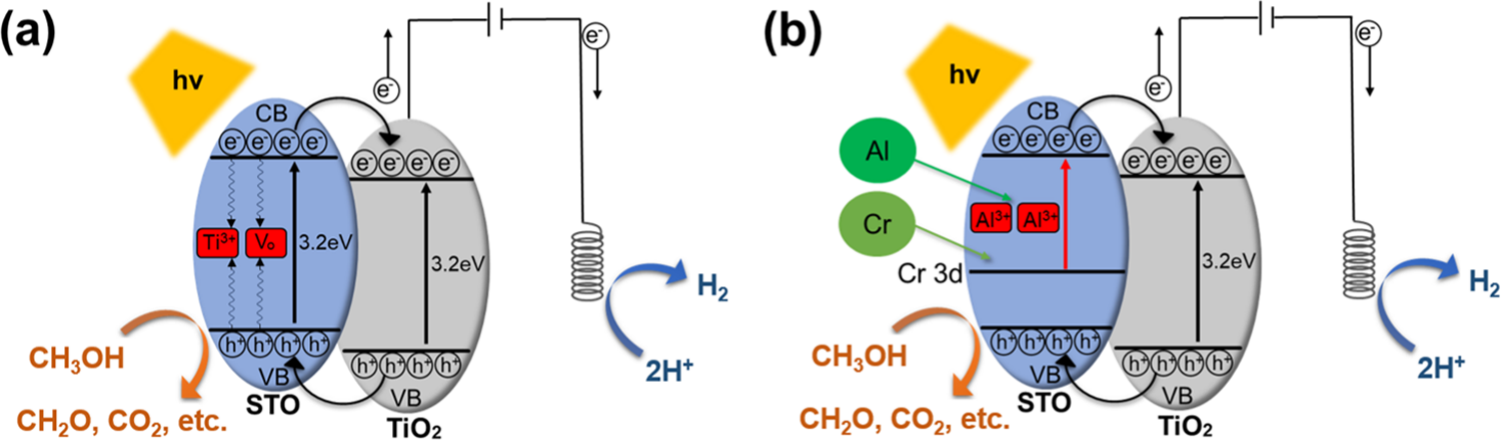
Illustration of possible reaction mechanisms during PEC measurements: (a) TNTA-STO and (b) TNTA-STO:Al,Cr.

**Figure 8:**
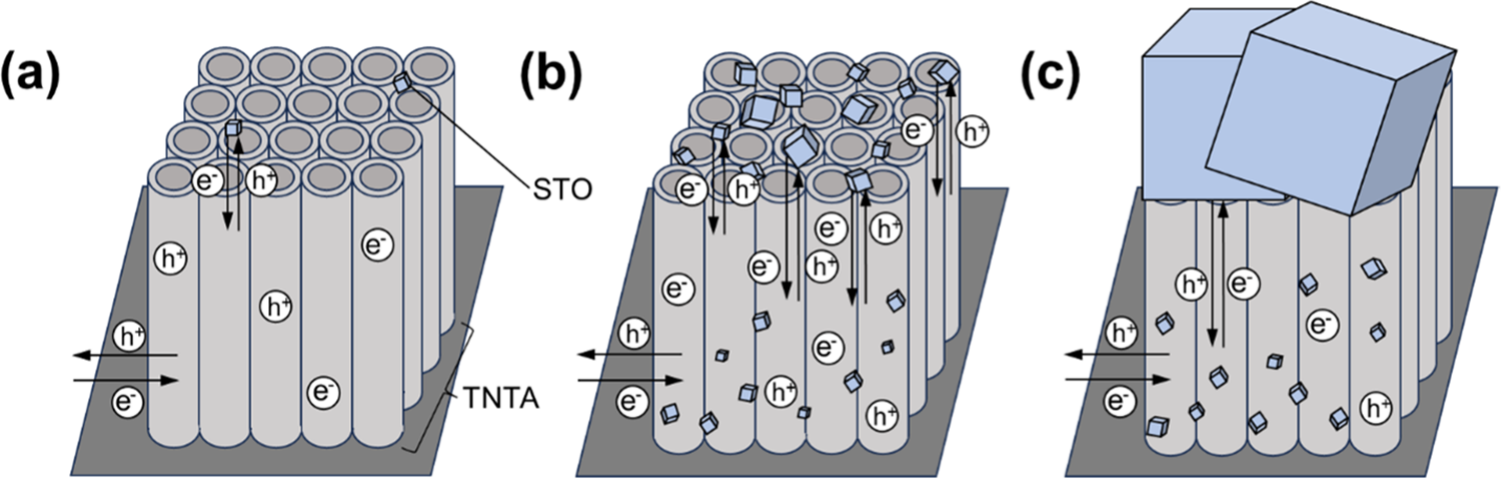
Illustration of photogenerated charge behavior in TNTA-STO:Al,Cr prepared by hydrothermal treatment for (a) 0.5 h, (b) 1 h, and (c) 1.5 h.

Figure 7 presents the possible PEC reaction mechanisms of TNTA-STO (a) and TNTA-STO:Al,Cr (b). In the case of TNTA-STO, although a type-II heterojunction is formed and the photogenerated charge separation is enhanced by the STO deposition, STO itself contains Ti^3+^ and oxygen vacancy (V_O_) levels within the band gap; therefore, considerable charge recombination in STO occurs. The introduction of Al^3+^ into STO serves to decrease the population of Ti^3+^ and V_O_, which work as charge recombination centers.^24^ The Cr doping results in the formation of Cr 3d levels in the band gap, and the band gap energy is reported to be narrowed from 3.2 to 2.3 eV.^21,36^ Notably, the band gap energy of 2.3 eV aligns with the wavelength of visible light, which suggests an improved response to visible light. In our sample, the light absorbance in the visible region was increased by the doping of Cr^3+^ (see Figure S3). This improvement is attributed to the formation of donor levels in the presence of Cr^3+^. To summarize, the results indicate that Al doping reduces the recombination rate by decreasing the presence of Ti^3+^ and V_O_ as the recombination center. On the other hand, Cr doping enhances the responsivity to visible light by creating donor levels and narrowing the band gap of STO to match the energy to visible light. Figure 8 is an illustration of TNTA-STO:Al,Cr under irradiation of the sunlight. In Figure 8a, less charge transfer occurs between STO and TiO_2_ than Figure 8b because of the smaller amount of STO, and many electrons and holes are recombined within defective STO. On the other hand, in Figure 8c, as the pores of TNTA are partially closed by the large STO, the active surface area becomes smaller than Figure 8b, which leads to the higher charge transfer resistance between photoanode and electrolyte. Therefore, the hydrothermal treatment for 1 h resulted in the deposition of moderate amount and size of STO and was the best to achieve the highest PEC performance.

## 4 Conclusions

In this study, experiments were conducted to elucidate the effect of Al and Cr codoping and the mechanism associated with the optimization of the hydrothermal treatment time. TNTA, TNTA-STO, and TNTA-STO:Al,Cr were prepared as photoanodes by utilizing anodization and successive hydrothermal treatment. The amount and size of STO on the TNTA surface were systematically varied by changing the duration of the hydrothermal treatment. The PEC characterization showed that TNTA-STO:Al,Cr_1 h possessed the best PEC properties under both simulated sunlight and visible light irradiation. Codoping of Al and Cr to STO enhanced the PEC performance, presumably due to the type II heterojunction formation, less defect in STO, and added visible light response. Too small STO deposition resulted in lowering the PEC performance, resulting from the low crystallinity, leading to higher charge recombination. Too much STO closed the tubular pores of TNTA, so deteriorated PEC performance was observed because of the lower active surface area. The hydrothermal treatment time of 1 h resulted in the deposition of an appropriate amount and size of STO codoped with Al and Cr on the TNTA surface, which exhibited the best PEC performance.

## Supporting Information Available

Raman spectra, energy-dispersive X-ray spectra, UV–visible spectra, and additional LSV curves (PDF)
