# Supplementary material for: Enhanced Photoelectrochemical Property of TiO2 Nanotube Array Photoanode Deposited with Al,Cr-Codoped SrTiO3 Nanocubes
Source: ACS Omega. 2024 Jan 3;9(2):2795–802. doi: 10.1021/acsomega.3c08014 (PMC10795150; doi:10.1021/acsomega.3c08014)
Supplement: Supplementary file 1 — ao3c08014_si_001.pdf [file ao3c08014_si_001.pdf]

Supporting Information

Enhanced photoelectrochemical property of TiO_2 nanotube array photoanode deposited with Al,Cr-codoped $SrTiO_3$ nanocubes

Shiho Hamazaki, Kazuki Inoue, Atsunori Matsuda, and Go Kawamura*

Department of Electric and Electronic Information Engineering, Toyohashi University of Technology, Toyohashi, Aichi 441-8580, Japan

Email: kawamura.go.km@tut.jp

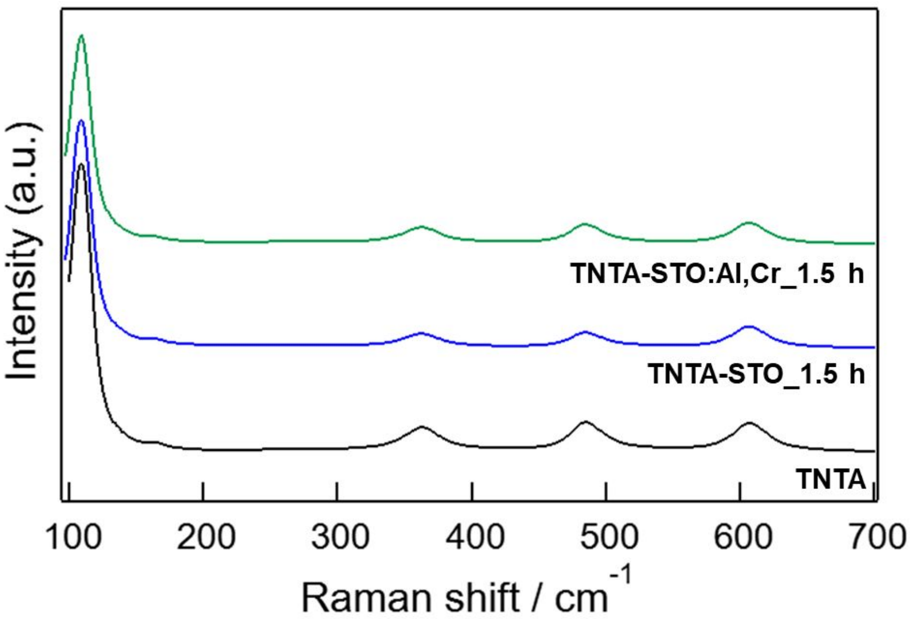

Fig. S1. Raman spectra of TNTA, TNTA-STO_1.5h, and TNTA-STO:Al,Cr_1.5h.

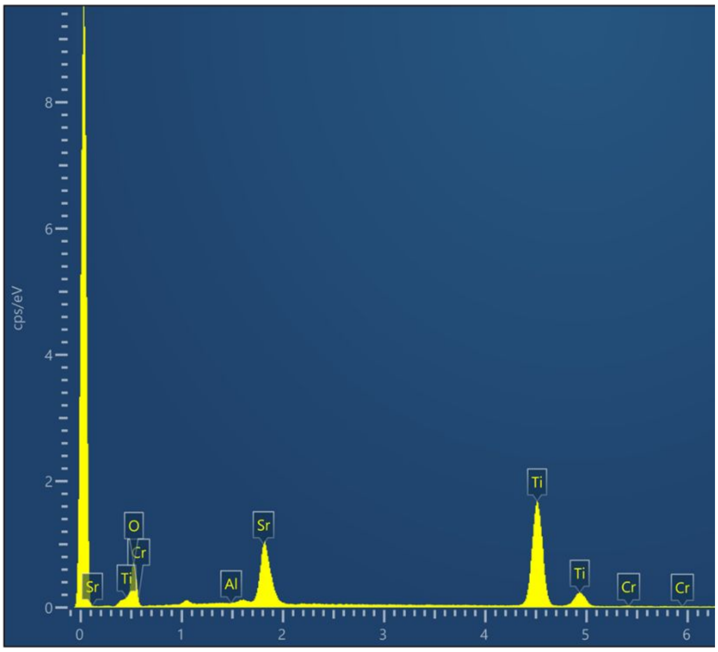

Sample	O	Ti	Sr	Al	Cr
TNTA-STO:Al,Cr_1.5h	70.0	23.7	6.3	0.0	0.1

Fig. S2. EDX data of TNTA-STO:Al,Cr_1.5h.

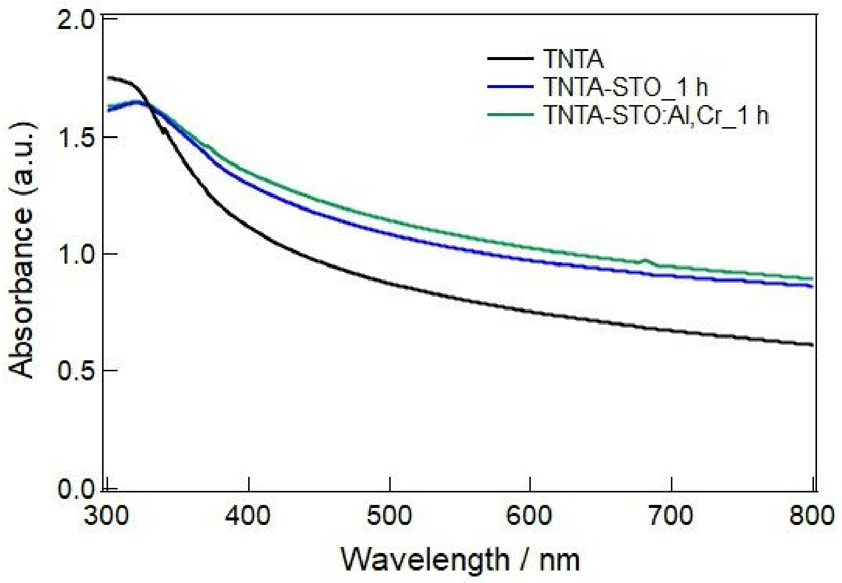

Fig. S3. UV-visible spectra of TNTA, TNTA-STO_1h, and TNTA-STO:Al,Cr_1h. The samples were scratched out from the Ti substrates and dispersed in water to measure the absorption spectra. A V-670 spectrophotometer (JASCO, Japan) was used.

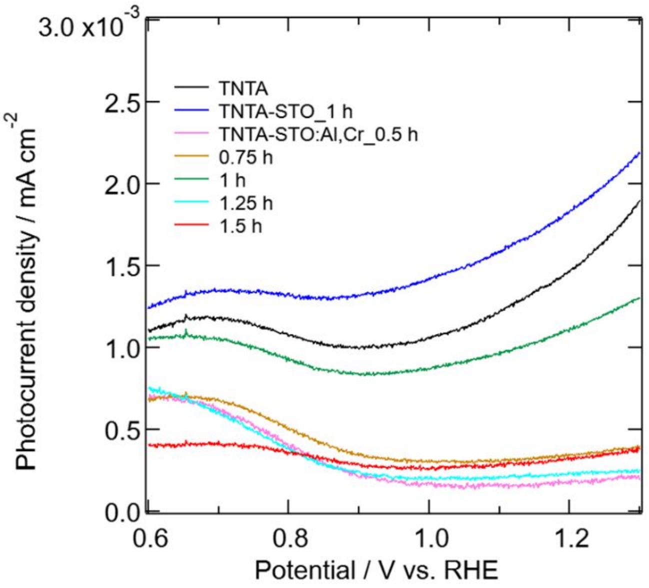

Fig. S4. LSV of prepared samples under dark condition.

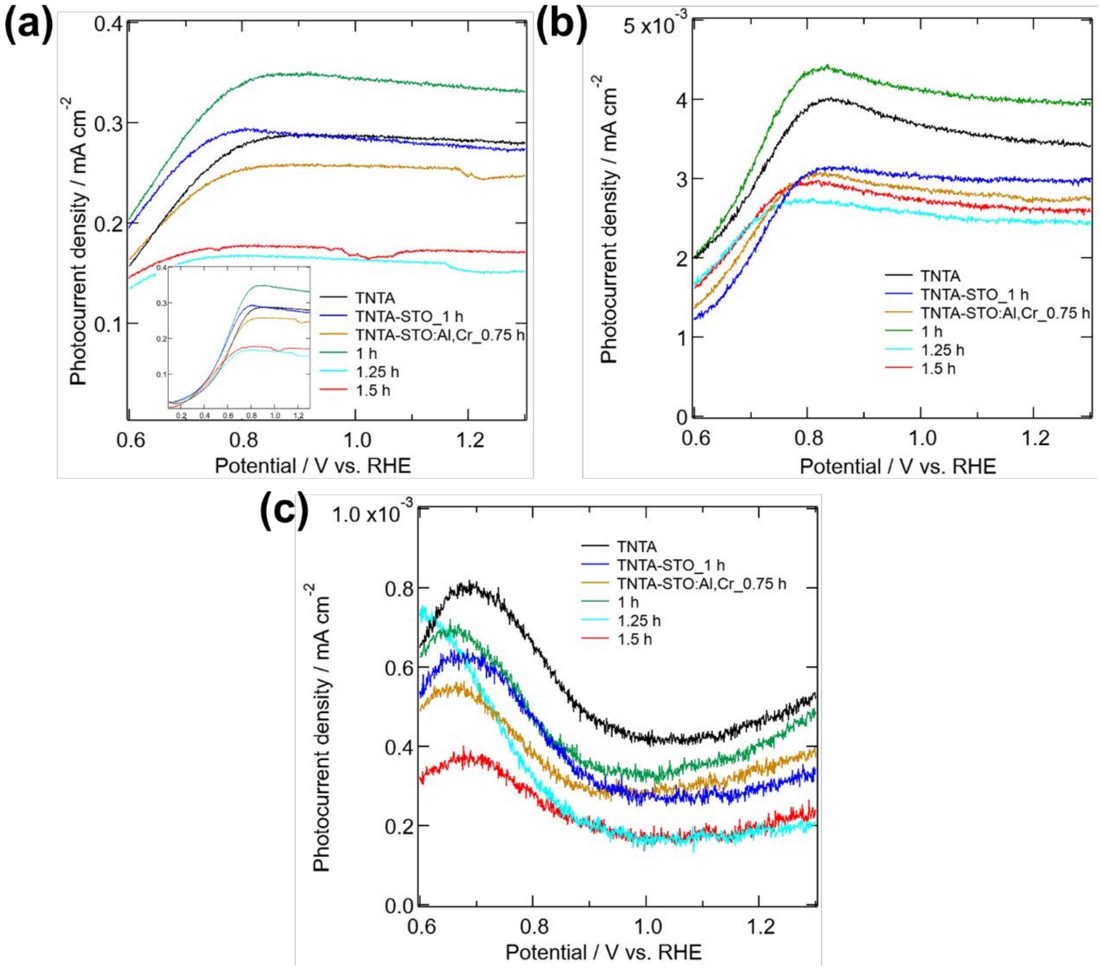

Fig. S5. LSV of prepared samples under (a) simulated sunlight, (b) visible light, and (c) dark condition (without methanol in the electrolyte).
